# All‐Scale Hierarchical Structure Contributing to Ultralow Thermal Conductivity of Zintl Phase CaAg_0.2_Zn_0.4_Sb

**DOI:** 10.1002/advs.202100109

**Published:** 2021-04-10

**Authors:** Jie Chen, Wenhua Xue, Chen Chen, Hongxing Li, Canying Cai, Qian Zhang, Yumei Wang

**Affiliations:** ^1^ Beijing National Laboratory for Condensed Matter Physics Institute of Physics Chinese Academy of Science Beijing 100190 P. R. China; ^2^ Department of Materials Science and Engineering and Institute of Materials Genome & Big Data Harbin Institute of Technology Shenzhen 518055 P. R. China; ^3^ School of Physics and Optoelectronics Xiangtan University Xiangtan 411105 P. R. China; ^4^ School of Materials Science and Engineering Xiangtan University Xiangtan 411105 P. R. China

**Keywords:** multidimensional defects, spherical aberration‐corrected electron microscopy, ultralow thermal conductivity, zintl phase

## Abstract

TiNiSi‐type Zintl phase CaAgSb can transform into LiGaGe‐type Zintl phase CaAg*_x_*Zn_(1−_ *_x_*_)/2_Sb when some of the Ag atoms are substituted by Zn atoms, leading to an ultralow thermal conductivity of ≈0.4 W m^−1^ K^−1^ in the whole measured temperature range of CaAg_0.2_Zn_0.4_Sb. The microstructure is then investigated by spherical aberration‐corrected electron microscopy on an atomic scale, which reveals an all‐scale hierarchical structure that can scatter the phonons in a wide frequency range. There exist a large quantity of CaAgSb nanometer precipitates as well as quite a lot of edge dislocations close to these nanometer precipitates, thus releasing the stress caused by the mismatch between the precipitates and the parent phase. Many twin boundaries also exist around the CaAgSb precipitates. High‐density point defects contain the randomly dispersed Ag vacancies and Zn atoms substituted for the Ag atoms. All these widely distributed multidimensional defects contribute to the decrease of lattice thermal conductivity in a wide temperature range.

## Graphical abstract

An ultralow lattice thermal conductivity (*κ*_L_) is measured to reach ≈0.4 W m^−1^ K^−1^ in the sample CaAg_0.2_Zn_0.4_Sb. The correlation is investigated between the microstructure and *κ*_L_ on an atomic scale using the *C*_s_‐corrected electron microscopy. It is revealed that the transport of phonons in a wide frequency range can be significantly impeded by an all‐scale hierarchical structure.

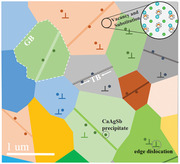

## 1 Introduction

Thermoelectric (TE) materials are attracting ever‐increasing attention due to the demand for clean energy in the world. Based on the Seebeck effect or the Peltier effect, TE materials are generally used for power generation or solid‐state refrigeration.^[^ ^1^, ^2^, ^3^, ^4^ ^]^ On the one hand, in thermoelectrics the charge carriers inside a material are used as a working medium; therefore, enabling a vibration‐free and emission‐free solution to the direct conversion between heat and electricity. On the other hand, the relatively low heat‐to‐power conversion efficiency restricts a large‐scale application of TE devices. To improve the performances of TE devices, it is pivotal to enhance the material's dimensionless figure‐of‐merit *ZT* = *σ* ^2^ *ST*/*κ*, where *S*, *σ*, *T*, and*κ*are the Seebeck coefficient, electrical conductivity, absolute temperature, and total thermal conductivity, respectively. The total thermal conductivity *κ* is composed of the lattice thermal conductivity (*κ*_L_) and the electronic thermal conductivity (*κ*_e_). It is a great challenge to simultaneously optimize the three parameters *S*, *σ*, and *κ * due to their inter‐related nature. Recently, there were suggested a number of new strategies for improving thermoelectric performance, such as establishing the Seebeck range to determine the optimization level of thermoelectric performance,^[^ ^5^ ^]^ manipulating the phonon dispersion to enhance the phonon scattering,^[^ ^6^ ^]^ introducing the superlattice precipitates to reduce thermal conduction,^[^ ^7^ ^]^ and inducing the splitting of band structures to optimize thermoelectric properties.^[^ ^8^ ^]^ The lattice thermal conductivity is relatively independent since it is related to the transport of phonons, instead of electrons. Thus, reducing the lattice thermal conductivity has been one of the most prevalent strategies to optimize the TE performance.^[^ ^9^, ^10^, ^11^, ^12^, ^13^, ^14^, ^15^ ^]^

Under the assumption of the ideal gas model, the lattice thermal conductivity of the crystal can be expressed as $\kappa_{L}=\frac{1}{3}C_{V}v_{g}^{2}\tau$, where *C*_V_ is the specific heat, *v*_g_ is the group velocity of the phonon vibration mode, and *τ* is the phonon relaxation time. To suppress the lattice thermal conductivity, we should search for a typical material with a lower value of *C*_V_ or *v*_g_.^[^ ^14^ ^]^ More prevailing efforts have been devoted to reducing the phonon relaxation time by strengthening the phonon scattering.^[^ ^14^ ^]^ Generally, nanostructures and defects are artificially designed. For example, 0D point defects, including substitutions,^[^ ^9^, ^16^ ^]^ interstitials,^[^ ^17^ ^]^ and vacancies^[^ ^18^, ^19^ ^]^ scatter the high‐frequency phonons. 1D dislocations^[^ ^12^, ^20^, ^21^, ^22^ ^]^ mostly scatter mid‐frequency phonons through the dislocation cores and strain. 2D interfacial scattering sources, including grain boundaries,^[^ ^23^, ^24^ ^]^ fine precipitates,^[^ ^10^ ^]^ modulations,^[^ ^25^, ^26^ ^]^ and inversion domain boundaries^[^ ^27^, ^28^ ^]^ effectively scatter low‐frequency phonons. To well understand the contribution of these defects to the phonon scattering, it is crucial to reveal the microstructural characteristics of thermoelectric materials on an atomic scale, which has been realized by using high‐resolution spherical aberration(*C*_s_)‐corrected scanning transmission electron microscopy (STEM) in many previous studies.^[^ ^27^, ^29^, ^30^, ^31^ ^]^ In particular, the results were successfully correlated with the transport behaviors,^[^ ^20^, ^24^, ^32^ ^]^ validating the capability of spherical aberration‐corrected electron microscopy in studying thermoelectric properties.

The TiNiSi‐type Zintl phase CaAgSb crystalizes into an orthorhombic structure with space group *Pnma*.^[^ ^33^ ^]^ An interesting structural transformation took place when the rare earth metal (RE = La, Ce, Pr, Nd, or Sm) atoms substituted for the Ca atoms, leading the band structure to be obviously modified and the TE properties to be significantly improved.^[^ ^33^ ^]^ A similar structural transformation was recently observed when Zn atoms replaced part of Ag atoms in CaAgSb. A new Zintl series CaAg*_x_*Zn_(1−_ *_x_*_)/2_Sb with *x* varying from 0.2 to 0.6 was obtained. These LiGaGe‐type compounds each have a hexagonal structure with space group *P*6_3_ *mc* and a remarkably lowered lattice thermal conductivity.^[^ ^34^ ^]^ Many other Zintl phase TE materials underwent this kind of structure transition with the doping concentration varying. For example, it has been reported that completely substituting Eu for Ca in Ca_9_Zn_4.5_Sb_9_ induces a structure to change from the orthorhombic structure (*Pbam*) into a hexagonal structure of Eu_2_ZnSb_2_ (*P*6_3_/*mmc*).^[^ ^27^ ^]^ Besides, a complex phase transition from *Pnma* to *P*6_3_ *mc* to *R*‐3*m* to *P*6_3_ *mc*, and finally to *Fm*‐3*m* happens with the doping content “*x*” increasing in Ca_1−_ *_x_*Ce*_x_*Ag_1−_ *_y_*Sb (0 ≤ *x* ≤ 1, 0 ≤ *y* ≤ 1).^[^ ^35^ ^]^

In this work, the CaAg_0.2_Zn_0.4_Sb is prepared by directly ball milling and hot pressing, and its ultralow lattice thermal conductivity is measured to reach ≈0.4 W m^−1^ K^−1^ within the whole measured temperature range, which conduces to the enhancement of *ZT* value. It is much higher than that of the CaAgSb.^[^ ^33^ ^]^ We investigate the correlation between the microstructure and the lattice thermal conductivity of this kind of Zintl phase material CaAg_0.2_Zn_0.4_Sb on an atomic scale by using the *C*_s_‐corrected electron microscopy. The subsequent structural investigation reveals that the transport of phonons in a wide frequency range can be significantly impeded by an all‐scale hierarchical structure.

## 2 Results

### 2.1 Details of Structure

**Figure 1:**
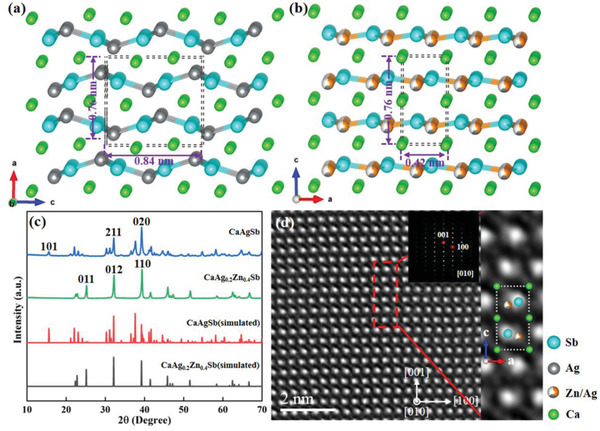
Atomic structure model of a) CaAgSb and b) CaAg_0.2_Zn_0.4_Sb. c) XRD patterns of CaAgSb and CaAg_0.2_Zn_0.4_Sb. d) *C*_s_‐corrected HAADF‐STEM image of CaAg_0.2_Zn_0.4_Sb, with inset exhibiting [010] SAED pattern.

**Figure 2:**
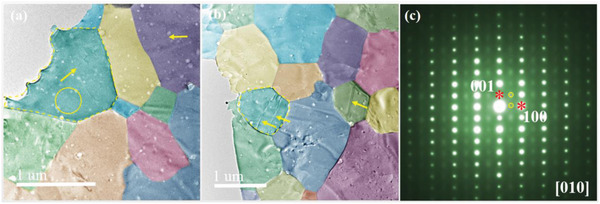
a,b) Low‐magnification TEM images of grains with different sizes in CaAg_0.2_Zn_0.4_Sb sample, with some strip contrasts indicated by arrows. c) [010] SAED pattern taken from circular region in (a).

Both CaAgSb and CaAg_0.2_Zn_0.4_Sb are Zintl phase compounds with complex crystal structures. **Figure** **1**a,b shows the structural transformation from *Pnma* (Figure 1a) to *P*6_3_ *mc* (Figure 1b). Single phase CaAg_0.2_Zn_0.4_Sb with a hexagonal structure is obtained (see Figure 1c). The energy dispersive spectrometer (EDS) results for CaAg_0.2_Zn_0.4_Sb are shown in Figure S1 in the Supporting Information. The four elements are evenly distributed in the CaAg_0.2_Zn_0.4_Sb and the actual composition obtained by EDS is Ca_0.955_Ag_0.195_Zn_0.4_Sb, which is very close to the nominal component. Figure 1d shows the *C*_s_‐corrected HAADF‐STEM image for CaAg_0.2_Zn_0.4_Sb, with the inset exhibiting the corresponding [010] selected area electron diffraction (SAED) pattern. Unlike the conventional TEM image, the HAADF‐STEM image has a contrast roughly proportional to *Z* ^1.7^, where *Z* is the atomic number. Hence the structural projection at the atomic level can be directly obtained. In the [010] direction, the atomic columns of Ca, Ag/Zn, and Sb are separately distinguishable as observed in Figure 1d, where the brightest dots represent the Sb atomic columns, the moderate ones denote the Ag/Zn atomic columns, and the weak ones refer to the Ca atomic columns. Some typical low‐magnification TEM images from different grains of the CaAg_0.2_Zn_0.4_Sb sample are shown in **Figure** **2**a,b, and the corresponding SAED pattern in Figure 2a (an area indicated by a yellow circle) is shown in Figure 2c. The CaAg_0.2_Zn_0.4_Sb compound is well crystallized with grain sizes ranging from 500 to 2000 nm. There exist some strips marked by arrows in these grains. These grains will be characterized in detail in Subsection 2.2. The SAED pattern (shown in Figure 2c) notably demonstrates two sets of electron diffraction spots. One is the main spots with the stronger intensity (indicated by stars), and the other is the weaker spots (denoted by circles). The main spots in the pattern can be indexed by the [010] orientation of the CaAg_0.2_Zn_0.4_Sb sample with the hexagonal structure (space group *P*6_3_ *mc*, *a* = 0.42 nm, *b* = 0.42 nm, and *c* = 0.76 nm). The origination of the weaker spots will be clarified from the high‐resolution images in the real space in Subsection 2.3.

### 2.2 Twin Boundary

**Figure 3:**
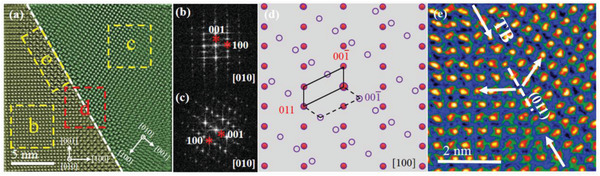
a) HAADF‐STEM image with sharp boundary indicated by the dashed line. b,c) FFTs for image regions (b) and (c) enclosed by dashed boxes in (a), respectively. d) Schematic view of superposition of (b) and (c). e) Magnified rectangular region (d) in (a).

An HAADF‐STEM image taken from the strip region in Figure 2a is shown in **Figure** **3**a. It can be seen from Figure 3a that there exists a difference in lattice orientation between its upper right part and lower left part. The sharp boundary is indicated by a dashed line. It is notable that the atomic arrangements in some regions near the boundary (for example, the region (e) enclosed in a dashed box) are different from those in other regions (for example, regions (b)–(d)). In order to ascertain the reason why the atomic arrangements around the boundary are different, the fast Fourier transform (FFT) is applied to both sides of the boundary, and the diffractograms from the selected regions b and c are respectively demonstrated in Figure 3b,c, which can be indexed by [010] of the CaAg_0.2_Zn_0.4_Sb. However, there is a 124° orientation difference between both sides of the boundary. The schematic view of a superposition of these two sets of the diffraction spots is shown Figure 3d, indicating that the twin boundary (TB) is situated in (011) plane. The magnified rectangular region d in Figure 3a is shown in Figure 3e, where atomically sharp and coherent TBs pointed by the arrows can be clearly observed. The measured angle between the crystal lattice on both sides of the boundary is about 124°, which accords with the qualitative analysis in the diffractograms as mentioned in Figure 3b,c. The presence of grain boundaries and TBs are beneficial to enhancing the long‐wavelength phonon scattering for a lowered lattice thermal conductivity in CaAg_0.2_Zn_0.4_Sb at lower temperatures.

### 2.3 Nanoprecipitate

**Figure 4:**
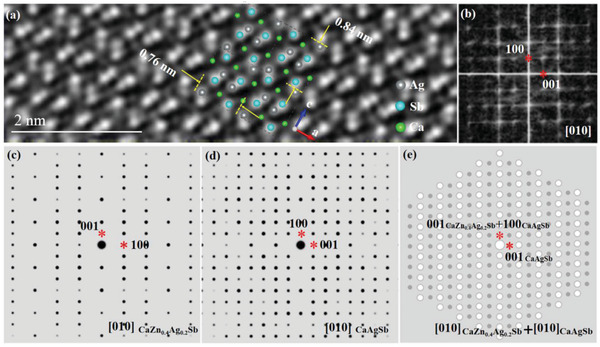
a) Magnification and b) FFT of rectangular region e in Figure 3a. [010] Simulated SAED patterns for c) CaAg_0.2_Zn_0.4_Sb and d) CaAgSb. e) Superposition of (c) and (d).

The atomic arrangement near the TB (see Figure 3a) is quite different from that in the [010] structural projection of CaAg_0.2_Zn_0.4_Sb as shown in Figure 1b. The magnified rectangular region e in Figure 3a (see **Figure** **4**a) shows that the minimum repetition unit is about 0.76 nm × 0.84 nm, which doubles in the [010] direction as great as that in the CaAg_0.2_Zn_0.4_Sb. The FFT (Figure 4b) corresponding to Figure 4a is well indexed by [010] of CaAgSb.^[^ ^33^ ^]^ Figure 4e shows the superposition of the [010] simulated diffraction patterns of CaAgSb (Figure 4c) and CaAg_0.2_Zn_0.4_Sb (Figure 4d), which matches well with the [010] diffraction pattern presented in Figure 2c. In addition, we also find the superposition of the diffraction patterns of the CaAgSb and CaAg_0.2_Zn_0.4_Sb in other diffraction orientations. For example, the experimental diffraction pattern shown in Figure S2a (Supporting Information) is the superposition of [1‐21] diffraction pattern of CaAg_0.2_Zn_0.4_Sb, together with [121] and [011] diffraction patterns of CaAgSb (the simulated diffraction patterns shown in Figure S2b–e in the Supporting Information). Hence, we can conclude that the stronger reflections correspond to the hexagonal structure of CaAg_0.2_Zn_0.4_Sb, and the weaker ones are caused by the orthogonal CaAgSb^[^ ^33^ ^]^ for the diffraction patterns shown in Figure 2c and Figure S2a (Supporting Information), suggesting the existence of a superposition of diffraction patterns from the parent phase CaAg_0.2_Zn_0.4_Sb and the precipitate CaAgSb. The HAADF‐STEM image (Figure 3a) provides the direct evidence that these two phases coexist in the form of microdomains. There is no sharp interface between the nanoprecipitate CaAgSb and the main phase. The CaAgSb nanostructure is probably the residual product when transforming CaAgSb into CaAg_0.2_Zn_0.4_Sb through Zn doping. The diffraction patterns of CaAgSb nanostructure are superimposed on those of CaAg_0.2_Zn_0.4_Sb with different orientations, leading to the complex diffraction patterns as shown in Figure 2c and Figure S2a (Supporting Information). The widely distributed CaAgSb nanoprecipitates very likely contribute to the effective phonon scattering.

### 2.4 Dislocation Defect

**Figure 5:**
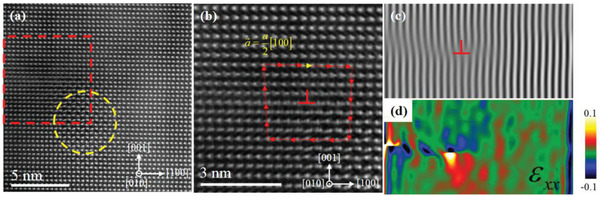
a) [010] HAADF‐STEM image of CaAg_0.2_Zn_0.4_Sb. b) The magnification of the rectangular region in (a). c) Filtered image of dislocation region in a), with inserted half plane indicated by symbol, ⊥. d) GPA of dislocation region in (a).

The nanometer precipitates actually exist everywhere in the CaAg_0.2_Zn_0.4_Sb sample, not just around twin boundaries. **Figure** **5**a shows a [010] HAADF‐STEM image for CaAg_0.2_Zn_0.4_Sb, where the CaAgSb precipitates and the lattice distortion induced by the dislocation are remarkable as indicated by the circle and rectangle, respectively. The magnified rectangular region including the dislocation is shown in Figure 5b. The Burgers vector is determined to be $\vec{a}=\frac{a}{2}\left[100\right]$ by making the closed clockwise Burgers circuit. In order to distinctly show the structure of the dislocation core, the filtering process for the rectangular region in Figure 5a is performed. In the filtered image shown in Figure 5c, the inserted half plane is pointed out by the symbol, ⊥. The Burgers vector $\vec{a}=\frac{a}{2}\left[100\right]$ is perpendicular to the direction of the dislocation line. Thus, the dislocation is an edge dislocation. The “Geometric Phase Analysis” plugin is introduced into DigitalMicrograph software to study the strain field around the dislocation core. The HAADF‐STEM image is analyzed via geometric phase analysis (GPA) as shown in Figure 5d, both of which could act as effective scattering centers for medium‐to‐long wavelength phonons. Another typical region for CaAg_0.2_Zn_0.4_Sb is also characterized by the HAADF‐STEM image as shown in Figure S3 (Supporting Information), where the lattice distortion induced by the dislocation is clearly displayed. So, there exist a large number of nanometer precipitates, as well as quite a lot of edge dislocations, which are close to these nanometer precipitates to release stress caused by the mismatch between the precipitates and the parent phase. Finally, we identify several different phonon scattering centers, such as TBs, CaAgSb nanometer precipitates, and edge dislocations, which very likely contribute to the lowering of lattice thermal conductivity within the whole temperature range.

### 2.5 Point Defect

**Figure 6:**
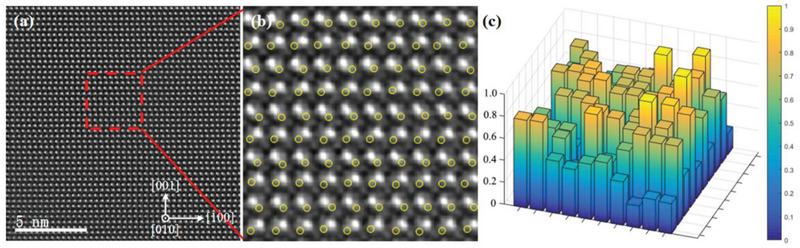
a) [010] HAADF‐STEM image of CaZn_0.4_Ag_0.2_Sb. b) The magnification of the rectangular region in (a). c) Statistical view of 3D intensity distribution for sites indicated by yellow circles in (b).

Finally, the point defects in the samples are investigated. These point defects are always considered to be beneficial to the high‐frequency scattering of the phonons in the TE materials.^[^ ^16^, ^17^, ^18^, ^19^ ^]^ **Figure** **6**a shows the [010] HAADF‐STEM image of the common area in CaAg_0.2_Zn_0.4_Sb. The rectangular region in Figure 6a is magnified and shown in Figure 6b. CalAtom software^[^ ^36^ ^]^ is used to calculate the intensity of the Ag/Zn/vacancy sites indicated by the yellow circles. A 3D stereogram is displayed in Figure 6c, indicating a disordered distribution of Ag atoms, Zn atoms, and vacancies. The structural transformation (from orthorhombic to hexagonal structure) results from the substitution of some Zn atoms for Ag atoms, such as CaAg_0.2_Zn_0.4_Sb. To balance the electronic state, one Zn atom replaces two Ag atoms, then leaving 40% occupancy at the Ag atom site. Therefore, the higher‐density point defects include the randomly distributed Ag vacancies and substitutions of Zn atoms for the Ag atoms.

## 3 Discussion

**Figure 7:**
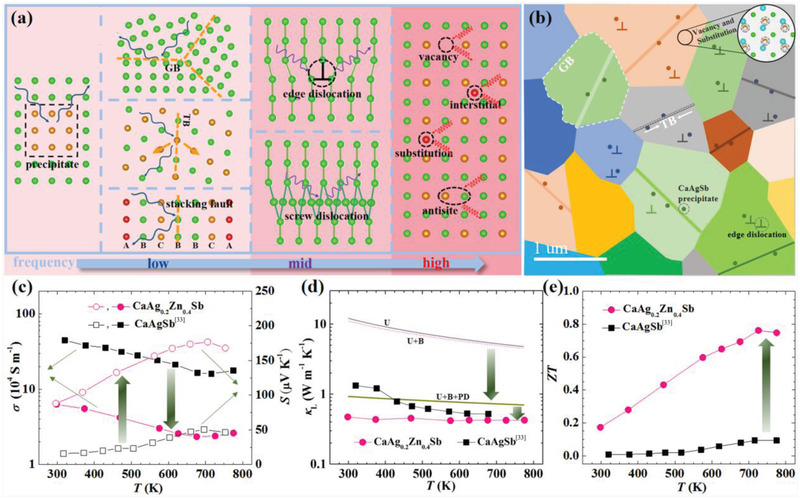
Illustration of a) defects frequently occurring in thermoelectric materials. b) Schematic view of microstructure in CaAg_0.2_Zn_0.4_Sb. Temperature dependence of c) electrical conductivity (left)/Seebeck coefficient (right), d) lattice thermal conductivity, and e) *ZT* value for CaAg_0.2_Zn_0.4_Sb and CaAgSb.^[^ ^33^ ^]^

A large number of defects ranging from 0D point defects to 3D nanoprecipitates, which are able to scatter phonons from high frequency to low frequency, are summarized in **Figure** **7**a. Fortunately, in CaAg_0.2_Zn_0.4_Sb we find all‐scale hierarchical structures, and we illustrate their microstructures in Figure 7b. The grain sizes of CaAg_0.2_Zn_0.4_Sb range from 500 to 2000 nm, which can act as effective scattering centers for the low‐frequency phonons.^[^ ^23^ ^]^ In addition, a lot of TBs (indicated by the lines in Figure 7b) together with widely distributed nanoprecipitates CaAgSb (indicated by the dots in Figure 7b) also enhance the phonon scattering at low frequency.^[^ ^10^, ^24^ ^]^ For middle wavelength photons and long wavelength phonons, 1D line defects, including many edge dislocations near the nanoprecipitates where the stress caused by the lattice mismatch between the precipitates and the parent phase is released, are believed to be workable.^[^ ^12^, ^20^, ^21^, ^22^ ^]^ Finally, a large number of disordered vacancies and substitutions (see the inset in the upper right corner of Figure 7b) become the excellent scatter centers for high‐frequency phonons.

Temperature‐dependent electrical conductivity, Seebeck coefficient, and total thermal conductivity for CaAg_0.2_Zn_0.4_Sb are measured and presented respectively in Figure 7c (left), Figure 7c (right), and Figure S4 (Supporting Information) in comparison with those for CaAgSb. As presented in Figure 7c, CaAg_0.2_Zn_0.4_Sb and CaAgSb show similar conduction behaviors with the temperature increasing. The electrical conductivity first decreases, and then increases slowly after 673 K. It should be noted that the higher Seebeck coefficient is observed in CaAg_0.2_Zn_0.4_Sb, because its band structure shows a separation between the conduction band and valence band.^[^ ^33^, ^34^ ^]^ The thermal conductivity for CaAg_0.2_Zn_0.4_Sb and CaAgSb are shown in Figure S4a in the Supporting Information. The thermal conductivity of CaAgSb decreases with seems temperature increasing. However, the thermal conductivity of CaAg_0.2_Zn_0.4_Sb is much lower than that of CaAgSb, and to be temperature independent.

The lattice thermal conductivity is calculated from *κ*_L_ = *κ* − *LσT*, where *L* is the Lorenz number estimated based on the single parabolic band model.^[^ ^27^ ^]^ As shown in Figure 7d, the lattice thermal conductivity of CaAg_0.2_Zn_0.4_Sb is significantly lower than that of CaAgSb within the whole measured temperature range, reaching an ultralow value of ≈0.4 W m^−1^ K^−1^ in a temperature range from 300 to 773 K. To explore the possible mechanism for reducing the lattice thermal conductivity of CaAg_0.2_Zn_0.4_Sb, we estimate the lattice thermal conductivity by using the Callway model^[^ ^37^ ^]^

(1) $$k_{l}=\frac{k_{B}}{2\pi^{2}v}{\left(\frac{k_{B}T}{\hbar }\right)}^{3}\int_{0}^{\theta_{D}/T}\tau \left(x\right)\frac{x^{4}e^{x}}{{\left(e^{x}-1\right)}^{2}}dx$$

where *x* is defined as ℏ*ω*/*k_B_T*, *k_B_* is the Boltzmann constant, *ћ* is the reduced Plank constant, *ω* is the phonon angular frequency, *v* is the average phonon velocity, *θ*_D_ is the Debye temperature, and *τ* is the total relaxation time relating to the Umklapp process (U), grain boundary (B), twin‐boundary (TB), point‐defects (PD), dislocations (D), and nanometer‐scale precipitate (P) scattering, and expressed as^[^ ^38^, ^39^, ^40^, ^41^, ^42^, ^43^, ^44^ ^]^

(2) $$\tau^{-1}=\tau_{U}^{-1}+\tau_{B}^{-1}+\tau_{\mathrm{TB}}^{-1}+\tau_{\mathrm{PD}}^{-1}+\tau_{D}^{-1}+\tau_{P}^{-1}$$

The relaxation time for Umklapp, grain boundary, and point defects scattering are given as follows

(3) $$\tau_{U}^{-1}\approx \frac{\hbar \gamma^{2}}{Mv_{a}^{2}\theta_{D}}\omega^{2}T\exp\left(-\theta_{D}/3T\right)$$

(4) $$\tau_{B}^{-1}=\frac{v}{d}$$

(5) $$\tau_{\mathrm{PD}}^{-1}=\frac{V\omega^{4}}{4\pi v_{a}^{3}}\Gamma_{M}\omega^{4}$$

where *γ* is the Gruneisen anharmonicity parameter, *M* is the average mass of an atom in the crystal, *v*_a_ is the average sound velocity, *d* is average grain size, *V* is the volume per atom, and *Γ*_M_ is the disorder scattering parameter.

Accroding to the above formulae and the parameters in Table S1 in the Supporting Information, we calculate the lattice thermal conductivity of CaAg_0.2_Zn_0.4_Sb. When only Umklapp and grain boundary scattering are considered, the estimated temperature‐dependent lattice thermal conductivity is obviously higher than the experimental result as shown in Figure 7d. After the point defect scattering is taken into consideration, the calculated lattice thermal conductivity significantly decreases to a value lower than 1 W m^−1^ K^−1^. It should be noted that the calculated results are still much higher than the experimental data. The further decreasing of lattice thermal conductivity is due mainly to the twin‐boundary, dislocations, and nanometer‐scale precipitates.

Combined with the lower thermal conductivity and the enhanced Seebeck coefficient, the *ZT* value of CaAg_0.2_Zn_0.4_Sb is dramatically improved within the whole temperature range from 300 to 773 K compared with that of CaAgSb. A peak *ZT* ≈ 0.8 is obtained at about 773 K, indicating the important contributions of all‐scale hierarchical structures in this promising material.

## 4 Conclusions

In this work, we have prepared CaAg_0.2_Zn_0.4_Sb by doping Zn at the Ag sites of CaAgSb. An ultralow lattice thermal conductivity ≈ 0.4 W m^−1^ K^−1^ is obtained in the whole measured temperature range, contributing to an enhanced *ZT* value compared with that of CaAgSb. Using the *C*_s_‐corrected electron microscopy, we investigate the microstructure of the sample on an atomic scale. A large number of defects with different dimensions, such as 0D point defects, 1D dislocations, 2D grain boundaries/twin boundaries and 3D nanoprecipitates are observed, which are responsible for suppressing the phonon transport in a large frequency range. Our results advance the understanding of phonon transport in Zintl Phase by illuminating the phonon scattering mechanism, and thus shedding a light on the subsequent property optimization.

## 5 Experimental Section

Calcium (Ca, 99.9%, shots), silver (Ag, 99.9%, shots), zinc (Zn, 99.999%, powders), and antimony (Sb, 99.999%, shots) were weighed according to the stoichiometry of CaAg_0.2_Zn_0.4_Sb, and then loaded into a stainless‐steel jar in an argon‐filled glove box. The mixtures were ball‐milled continuously for 10 h by using a high energy ball mill (SPEX 8000M). The obtained powder was sintered into a dense disk at 853 K for 2 min by using the spark plasma sintering (SPS) technology under an axial pressure of 60 MPa.

Specimens used for transmission electron microscopy (TEM) observation were prepared by traditional mechanical polishing, dimpling, and then ion milling with liquid nitrogen stage. TEM and high‐angle annular dark field (HAADF)‐STEM investigations were carried out by using a JEM‐ARM 200F equipped with a cold FEG source and double‐*C*_s_ correctors operated at 200 kV. The attainable spatial resolution of the microscope was 80 pm. The HAADF images were acquired at acceptance angles in a range of 70–150 mrad. All the HAADF images presented in this work were Fourier‐filtered to reduce the influence of irregular noise.

The Seebeck coefficient (*S*) and electrical conductivity (*σ*) were simultaneously measured on a commercial apparatus (CTA‐3, CRYALL) and double checked on another commercial apparatus (ZEM‐3, ULVAC). The thermal conductivity (*κ*) was calculated from *κ* = *DαC*_p_, where *D* is the volumetric density determined by the Archimedes method, *α* is the thermal diffusivity measured on a laser flash apparatus (Netzsch LFA457), and *C*_p_ is the specific heat obtained by using a differential scanning calorimetry thermal analyzer (Netzsch DSC 404 F3). The longitudinal (*v*_L_) and transverse (*v*_T_) component of the sound velocity were measured by using an ultrasonic pulse receiver (Olympus) equipped with an oscilloscope (Tek‐tronix).

CalAtom software^[^ ^36^ ^]^ was used for the statistical analysis of the Ag/Zn/vacancy. Intensity of the Ag/Zn/vacancy sites was normalized and presented in Subsection 2.5.

## Conflict of Interest

The authors declare no conflict of interest.

## Supporting information

Supporting Information

## Data Availability

Research data are not shared.
