# Supplementary material for: All‐Scale Hierarchical Structure Contributing to Ultralow Thermal Conductivity of Zintl Phase CaAg0.2Zn0.4Sb
Source: Adv Sci (Weinh). 2021 Apr 10;8(11):2100109. doi: 10.1002/advs.202100109 (PMC8188219; doi:10.1002/advs.202100109)
Supplement: Supplementary file 1 — Supporting Information [file ADVS-8-2100109-s001.pdf]

© 2021 Wiley-VCH GmbH

ADVANCED SCIENCE
Open Access

Supporting Information

for *Adv. Sci.,* DOI: 10.1002/advs.202100109

All-scale Hierarchical Structure Contributing to Ultra-low Thermal Conductivity of Zintl Phase $CaAg_{0.2}Zn_{0.4}Sb$

Jie Chen[1,3], Wenhua Xue[1,2], Chen Chen[2], Hongxing Li[3], Canying Cai[4], Qian Zhang[2], and Yumei Wang[1*]*

All-scale Hierarchical Structure Contributing to Ultra-low Thermal Conductivity of Zintl Phase $CaAg_{0.2}Zn_{0.4}Sb$

Jie Chen[1,3], Wenhua Xue[1,2], Chen Chen[2], Hongxing Li[3], Canying Cai[4], Qian Zhang[2*], and Yumei Wang[1*]

[1]Beijing National Laboratory for Condensed Matter Physics, Institute of Physics, Chinese Academy of Science, Beijing 100190, P.R.China

[2]Department of Materials Science and Engineering and Institute of Materials Genome & Big Data, Harbin Institute of Technology, Shenzhen 518055, P.R.China

[3]School of Physics and Optoelectronics, Xiangtan University, Xiangtan 411105, P.R.China

[4]School of Materials Science and Engineering, Xiangtan University, Xiangtan 411105, P.R.China

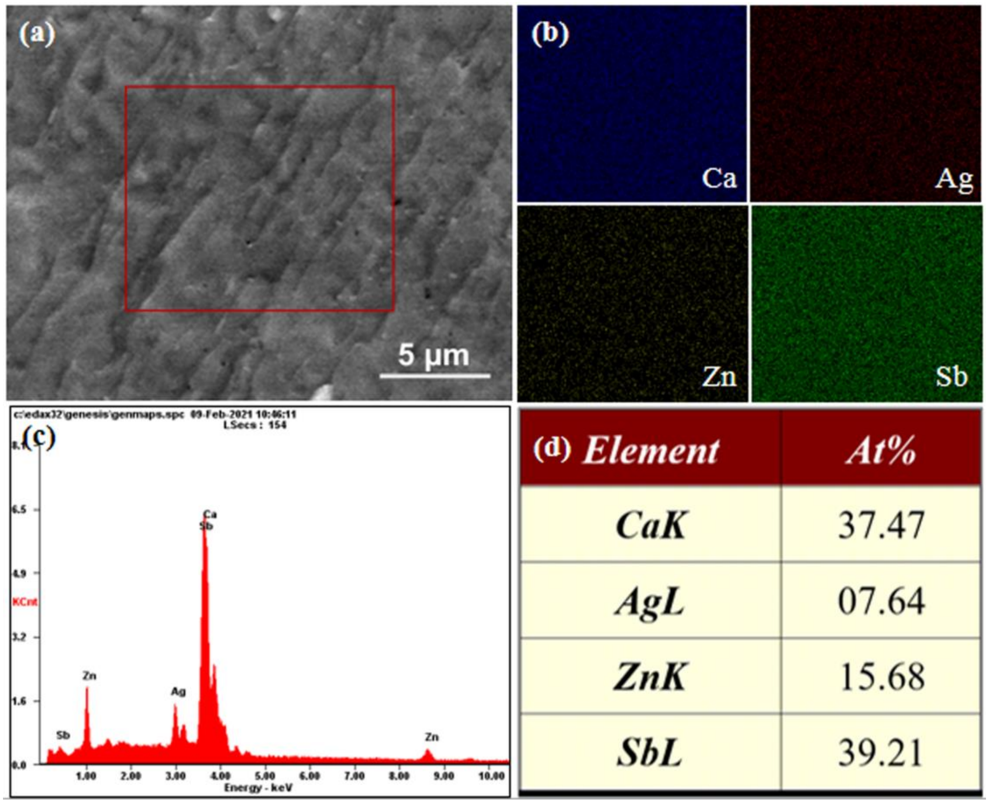

Figure S1. (color online) a) SEM image of $CaAg_{0.2}Zn_{0.4}Sb$. b, c) EDS analysis of rectangular region in a). d) Percentages of elements in $CaAg_{0.2}Zn_{0.4}Sb$.

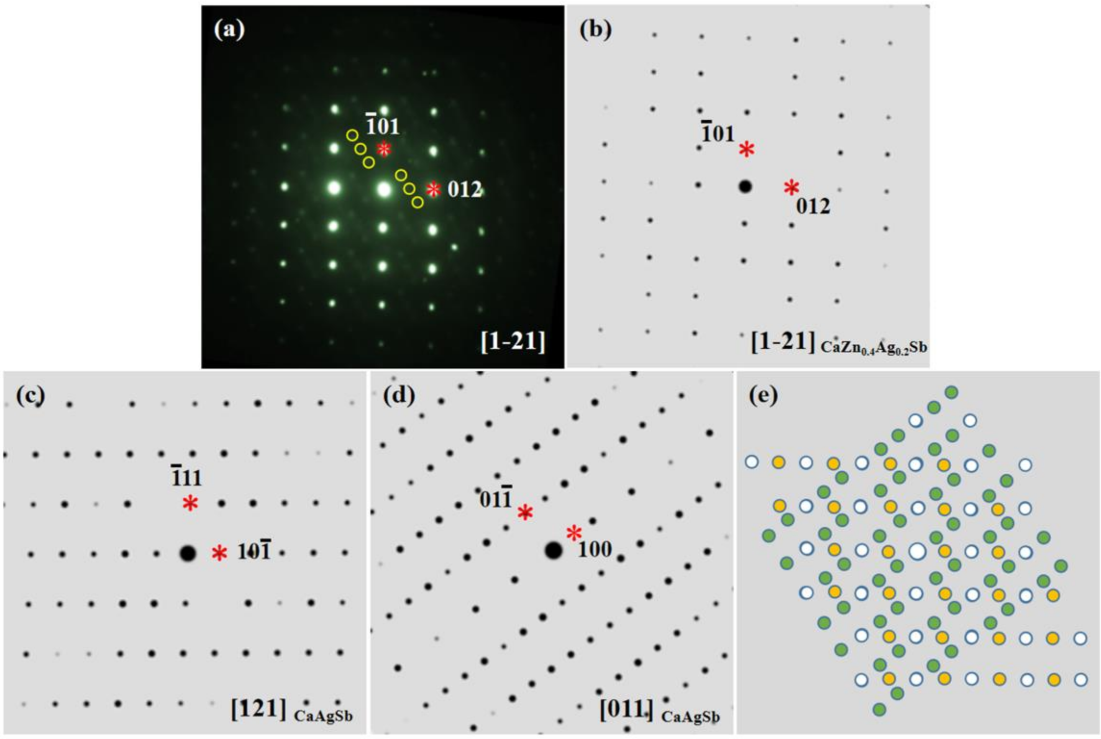

Figure S2. (color online) a) SAED pattern of $CaZn_{0.4}Ag_{0.2}Sb$. Simulated SAED patterns of b) [1-21] for $CaZn_{0.4}Ag_{0.2}Sb$, c) [121] for CaAgSb, and d) [011] for CaAgSb. e) Superposition of b), c) and d).

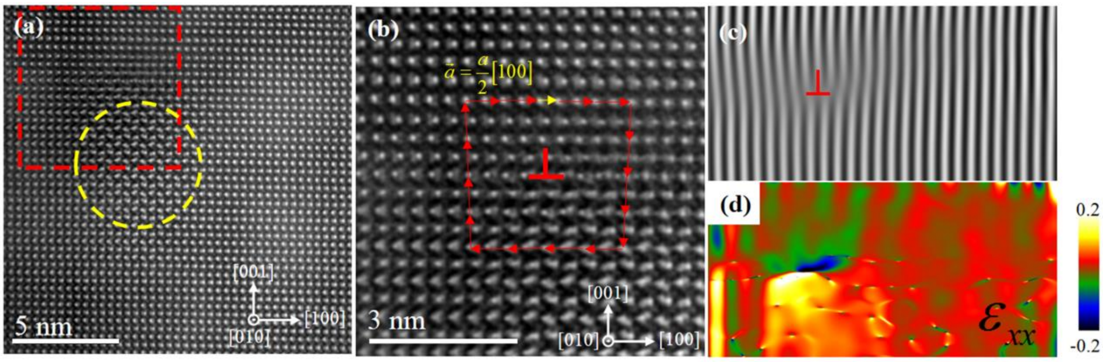

Figure S3. (color online) a) [010] HAADF-STEM image of $CaZn_{0.4}Ag_{0.2}Sb$. b) The magnification of the rectangular region in a). c) Filtered image of dislocation region in a), with inserted half plane indicated by symbol, ⊥. d) GPA of dislocation region in a).

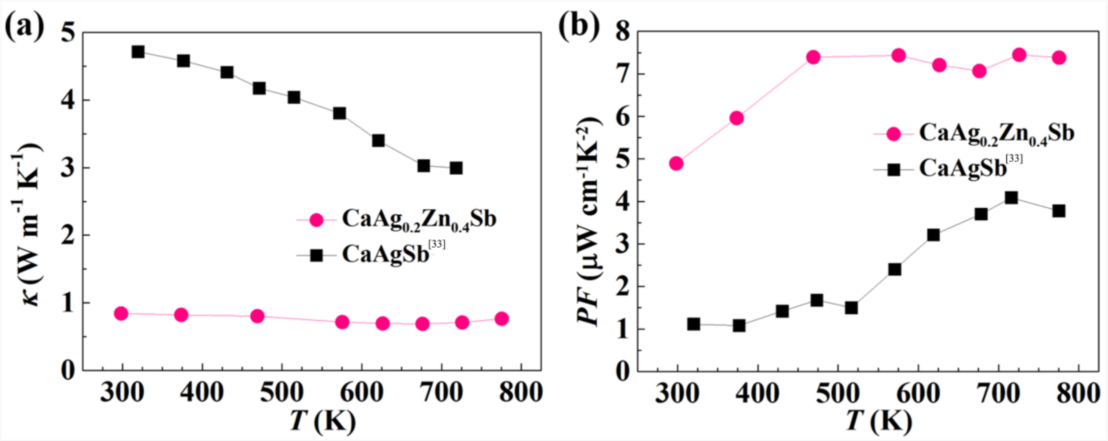

Figure S4. (color online) Temperature dependence of a) total thermal conductivity and b) power factor for $CaAg_{0.2}Zn_{0.4}Sb$ and CaAgSb,[33] respectively.

V_L (m/s)	4078	V_T (m/s)	2272
Young's modulus, E (GPa)	64.36	Average sound velocity, v_a(m/s)	2529
Shear modulus, G (GPa)	25.34	Average relative atomic mass	80.62
Poisson ratio, v_p	0.27	Average mass --M	1.338×10^{-25}
Gruneisen parameter, γ	1.6	The average volume/atom	2.737×10^{-29}
Debye temperature, θ_D (K)	258.4	Density (kg m^{-3})	4890

Table S1. Parameters taken from our calculations and measurements for calculating lattice thermal conductivity.
